# The Increasing Trend of Cesarean Section in Iran: A Challenge for the Health System

**DOI:** 10.34172/aim.34028

**Published:** 2025-05-01

**Authors:** Habibollah Azarbakhsh, Rozhan Khezri, Seyed Parsa Dehghani, Fatemeh Rezaei

**Affiliations:** ^1^Department of Epidemiology, Faculty of Health, Ahvaz Jundishapur University of Medical Sciences, Ahvaz, Iran; ^2^Student Research Committee, Iran University of Medical Sciences, Tehran, Iran; ^3^School of Medicine, Shiraz University of Medical Sciences, Shiraz, Iran; ^4^Research Center for Social Determinants of Health, Jahrom University of Medical Sciences, Jahrom, Iran

## To Editor,

 Caesarean section has an important role in saving the life of the mother and the baby, but it is recommended only in special medical conditions. According to the available information, 21.1% of births worldwide are occur by caesarean section, although the rate varies in different regions of the world.^1^ Iran has one of the highest caesarean section rates in the world. From March 21, 2019 to March 21, 2021, it was reported that 2 322 500 women gave birth, of whom 53.6% delivered through Caesarean section.^2^ This is despite the fact that according to the recommendations of the World Health Organization, the caesarean rate above 20% does not improve perinatal results.^3^

 In Iran, the rate of caesarean section is very high which is an alarm requiring attention to the amount of non-medical caesarean sections. Failure to pay attention to this increasing trend of caesarean section in Iran can cause adverse effects by resulting in adverse consequences for the health of the mother and baby. Therefore, it is vital to develop appropriate strategies and perform timely interventions to prevent unnecessary caesarean sections in Iran. If this trend continues, caesarean sections will impose a heavy financial burden on societies and health systems. So, the health system staff must provide comprehensive health education and assuage the fear and anxiety of pregnant women, relieve pain, explain the advantages and disadvantages of caesarean section, engage in respectful communication, emotional support, and transparency of the risks to ensure a vaginal birth with safer results and provide positive motherhood experiences and lower costs.^4^ It is suggested to identify the groups that have the greatest impact on the overall rate of cesarean section, so that if possible, effective strategies and necessary interventions can be applied to reduce the rate of cesarean delivery in these groups. It is also recommended to carry out qualitative and quantitative studies with the aim of determining the reasons for the desire of pregnant women to perform a cesarean section in unnecessary cases.

## References

[1] Boerma T, Ronsmans C, Melesse DY, Barros AJD, Barros FC, Juan L (2018). Global epidemiology of use of and disparities in caesarean sections. Lancet.

[2] Pourshirazi M, Heidarzadeh M, Taheri M, Esmaily H, Babaey F, Talkhi N (2022). Cesarean delivery in Iran: a population-based analysis using the Robson classification system. BMC Pregnancy Childbirth.

[3] World Health Organization Human Reproduction Programme (2015). WHO Statement on caesarean section rates. Reprod Health Matters.

[4] The Lancet (2018). Stemming the global caesarean section epidemic. Lancet.

